# Formulation and Mathematical Optimization of Controlled Release Calcium Alginate Micro Pellets of Frusemide

**DOI:** 10.1155/2013/819674

**Published:** 2013-07-22

**Authors:** Amitava Ghosh, Prithviraj Chakraborty

**Affiliations:** Bengal College of Pharmaceutical Sciences & Research, Bidhannagar, Durgapur, West Bengal 713212, India

## Abstract

*Objective*. Frusemide loaded calcium alginate micropellets, an oral microparticulate delivery system, was statistically optimized exhibiting prolonged therapeutic action minimizing its adverse effects. *Methods*. Ionotropic Gelation technique was adopted employing 3^2^ Factorial designs and keeping the entire process free from organic solvents. Physicochemical and the release characteristics of the prepared formulations were studied, keeping variations only in sodium alginate (primary polymer) and Acrycoat E30D (copolymer) dispersion. *Result*. Sodium alginate was predominant over Acrycoat E30D in all batches. Nonadditives or interaction was observed to be insignificant. Multiple regressions produced second-order polynomial equation, and the predictive results obtained were validated with high degree of correlation. The *in vivo* study applauded that optimized calcium alginate micropellets of frusemide can produce a much greater diuretic effect over an extended period of 24 hours. *Conclusion*. This study reveals that the potential of a single dose of the mathematically optimized micro pellets of frusemide formulation is sufficient in the management of peripheral edema and ascites in congestive heart failure and as well in the treatment of chronic hypertension, leading to better patient compliance, and can be produced with minimum experimentation and time, proving far more cost-effective formulation than the conventional methods of formulating dosage forms.

## 1. Introduction 

The development of pharmaceutical formulation depends on several factors and process parameters. The response variables related to effectiveness, safety, and usefulness must be optimized through a factorial relationship by combining the casual factors. However, this effort addresses a multiobjective optimization problem since it has to circumvent many difficulties in the quantitative approach, like understanding of the actual relationship between casual factors and individual pharmaceutical responses or the prediction of those formulations that are desirable for as many as possible drug properties [1].

Due to the complex nature of the development of pharmaceutical formulations, some computer based optimization techniques have been proposed in the literature. Among them, factorial design (FD) and response surface methodology (RSM) are the most widely used, and several research efforts have adopted either FD followed by an RSM or solely RSM. FD is a technique that contributes to the structure of data collection process. Through a designed experiment, FD is capable of characterizing the relationship between important and unimportant factors. The aim of RSM is utilized to find out the ideal operating conditions for a given system or the manner in which a particular response is affected by a set of variables over some specific regions of interest. The first step in the RSM is to find out a suitable approximation of the true functional relationship between the dependent variable and the set of independent variables (factors) [1]. Based on the principal of design of experiments (DoE), the methodology uses various types of experimental design, generation of polynomial equations, and mapping of the response over the experimental domain to determine the optimum formulation(s) [2–6].

The technique requires minimum experimentation and time, thus proving far more cost-effective formulation than the conventional methods of formulating dosage forms.

The current study aims at developing and optimizing an oral micropellet system of calcium alginate containing frusemide (an anthranilic acid derivative and a loop diuretic used in the treatment of congestive heart failure and edema) using RSM, as it is expected to be more productive than the conventional CR systems by virtue of prolongation of drug residence time in GI tract. Computer-aided optimization technique, factorial 2FI Model, was employed to investigate the effect of the primary polymer, sodium alginate (sodium salt of alginic acid), and copolymer, Acrycoat E30D (containing 30% solids which is prepared by emulsion polymerization and consists of neutral copolymers of ethyl acrylate and methyl methacrylate esters that are insoluble over the entire physiological pH range), as the two important variables [7], on the nature and performance of the microparticulate drug delivery system. 

## 2. Materials and Methods

### 2.1. Materials

Frusemide was provided ex gratia by Aventis Pharma Ltd. (Ankleshwar, India), Acrycoat E30D was a gift from Corel Pharma (Ahmedabad, India), and sodium alginate was purchased from Loba Chemie, (Mumbai, India). Calcium chloride was purchased from RanChem (India). All other chemicals employed were of analytical grade.

### 2.2. Experimental Design

In order to optimize the formulation design in producing calcium alginate micropellets of frusemide the effects of the primary polymer, sodium alginate, and copolymer, Acrycoat E30D, on the nature and performance of the micro particulate drug delivery system were studied as two important variables. To get an estimate of the performance of the drug delivery system from the preliminary investigations [7], the following dissolution parameters were considered as responses of the variables.

- Zero-order release rate constant—*K*_0_ (mg/hr).
- Amount of drug released in 2 hours (burst effect)—*X*_120_ (mg).
- Time required for 80% drug release—*t*_80_ (hr).
- Peppas Diffusion coefficient—*n*.

From the preliminary *in vitro* dissolution aspect of the formulations, it has been seen that on a span of overall release of 0–9 hours the release mechanism of the drug followed predominantly zero-order release model [7]. Hence, the zero-order release rate constant (*K*_0_) was selected as viable response to be studied statistically. The values of *K*_0_ of the nine different formulations were analyzed by 2-way ANOVA and linear regression analysis. The regression equation obtained from the analysis was used to predict the value of *K*_0_ with uninvestigated concentrations of sodium alginate and Acrycoat E30D. Formulations with the said concentrations were then prepared, and the actual *K*_0_ value obtained was compared with the predicted value. The other responses studied in this investigation were as follows (*X*_120_) the amount in mg of drug released in 120 minutes or 2 hours was taken into account so as to have an estimate of burst release mechanism and initial therapeutic dose being made available by the formulations so as to elicit fast onset of action, (*t*_80_) the time required for the drug to release 80% of its actual content gives an indirect estimation of the dissolution efficiency of the formulations as calculations of 100% release would have been too ideal to study, and the diffusion coefficient factor (*n*) of Korsmeyer-Peppas equation signifies that the diffusion behaviour of the drug from the polymer matrix follows either Fickian or Non-Fickian mechanism [8]. Statistical optimization was necessary to justify the significance or non significance “*n*” irrespective of the concentration of the polymers. The design summary was depicted in Table 1.

### 2.3. The Formulation Design: Full 3^2^ Factorial Design

Micropellets of frusemide were prepared by full 3^2^ factorial designs. Only two variables, with variations at three levels, namely, high, medium, and low, were observed by varying the concentrations of Sodium alginate and Acrycoat E30D. Hence, 3^2^ model designs were selected. High, medium, and low levels of sodium alginate were 4% w/v, 2% w/v, and 1% w/v, respectively, and that for Acrycoat E30D the levels were 4% w/w, 2% w/w, and 0% w/w. All the other parameters were kept unchanged from its optimized level. The formulation design is tabulated in Table 2.

### 2.4. Preparations of Frusemide Loaded Calcium Alginate Micropellets

**Figure 1:**
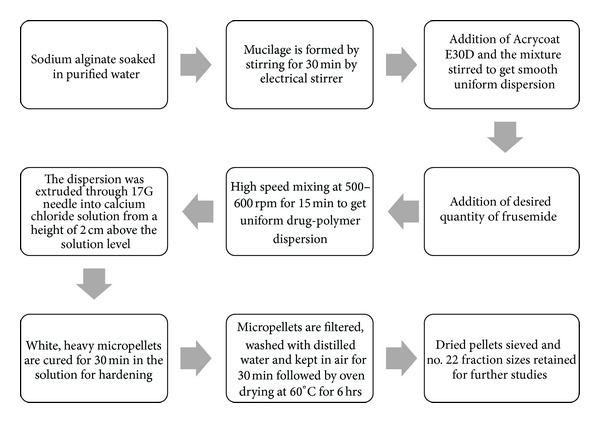
Flow sheet of the ionotropic gelation method employed for the preparation of frusemide loaded calcium alginate micropellets.

A flow sheet of the entire methodology for the preparation of frusemide loaded calcium alginate micropellets was shown in Figure 1.

### 2.5. Particle Size Analysis

A sample of fully dried micropellets (10 g) was placed on the top sieve no. 16. The set of Indian Pharmacopoeia standard [9] sieves were arranged in the order of nos. 16, 22, 30 of aperture size 1000, 710, and 500 *μ*m, respectively. The entire set was shaken for 5 minutes in a sieve shaker. Pellets retained in each sieve were weighed, and the percentage weight retained against various sieve size was recorded. The average particle size was calculated from the following formula [8]:

(1) $$\begin{array}{c} d_{\text{average}}=\frac{\Sigma \left(n\times d\right)}{\Sigma n}, \end{array}$$

where *n* is the frequency weight and *d* is the mean size. The latter was calculated by taking the average of the nominal mesh size of two corresponding sieves. The average diameter and the mode of distribution were calculated mathematically for six times, and the results were tabulated in Table 3.

### 2.6. Rheological Study

To assess the flow properties of the prepared micropellets measurement of angle of repose method [10] was employed. The angle of repose was calculated (Table 3) by measuring the height (*h*) of the pile and the radius of the base (*r*) with a ruler. The results obtained are recorded in triplicate.

### 2.7. Determination of Drug Content and Drug Entrapment Efficiency (DEE) of the Frusemide Loaded Calcium Alginate Micropellets

About 100 mg of micropellets (no. 30 sizes) was accurately weighed and dissolved in 25 mL of phosphate buffer (pH 7.4) and kept overnight. An aliquot from the filtrate was analysed spectrophotometrically, after suitable dilution, using SHIMADZU UV-VIS spectrophotometer, at 277.5 nm. Reliability of the method [11] was judged by conducting recovery analysis using known amount of drug with or without polymer. Recovery was found to be averaged at 100 ± 0.89%. Drug content of every batch was determined in triplicate for (no. 30) size range of micropellets, and the mean ± SD was calculated. Drug entrapment efficiency (DEE) was calculated (Table 3) using the formula

(2) $$\begin{array}{c} \%\text{DEE}=\left(\frac{\text{Actual}\;\;\text{drug}\;\;\text{content}}{\text{Theoretical}\;\;\text{drug}\;\;\text{content}}\right)\times 100. \end{array}$$

### 2.8. Loose Surface Crystal (LSC) Study

100 mg of micro pellets (no. 30 sizes) was suspended in 100 mL of phosphate buffer (pH 6.8), as the dissolution media. The samples were shaken vigorously for 15 min in a mechanical shaker. The amount of drug leached out from the surface was analysed spectrophotometrically, after suitable dilution, using SHIMADZU UV-VIS spectrophotometer at 277.5 nm. Percentage of drug present loosely on the surface of the micropellets was obtained (Table 3) using following equations [12].

- %LSC with respect to weight of micropellets = amount of drug (mg) released after 15 min/total weight of micropellets used in the experiment × 100.
- %LSC with respect to entrapped drug = amount of drug (mg) released after 15 min/drug content of micropellets used in the experiment × 100.

### 2.9. Disintegration Study

Disintegration study was performed in 0.1 M hydrochloric acid and USP phosphate buffer pH 6.8 separately in a rotating bottle apparatus [11]. 5 Micropellets of a fixed size fraction were rotated in 50 mL of the liquid medium in glass vial at 37°C at 30 rpm for 2 hours. Tests were performed in triplicate. Disintegration time (min) was observed (Table 3) when the micropellets were swelled and finally disintegrated and dispersed in the medium. No swelling occurred in 0.1 M hydrochloric acid over 24 hours. 

### 2.10. Study on Drug: Polymer Interaction Using Infrared (IR) Spectroscopy

The method adopted was Disc Method as per British Pharmacopoeia [13]. The drug and formulation were grounded carefully, spread uniformly in a suitable die, and submitted *in  vacuo* to a pressure of about 800 MPa (8 t·cm^−2^). Disc samples were analysed in FTIR spectrophotometer (SHIMADZU FTIR-8400S, Japan) over a range of 400–4000 cm^−1^. Both absorbance (*A*) and transmittance (*T*) spectra were recorded.

### 2.11. Study on Drug: Polymer Interaction Using High Performance Liquid Chromatography (HPLC)

The primary objective of the study was to identify any potential interactions or incompatibility among the drug and the polymers used in the formulation. The principle behind this identification was by comparing the retention time of both the standard drug sample and the drug extracted from the micropellets containing all the polymers [14]. The value of *R*_*r*_ ~ 1 signifies nil or insignificant interaction. 10 *μ*L of the sample was injected (HPLC: SHIMADZU LC-20AT/SPD-20A, JAPAN) and detected at a wavelength of 277.5 nm.

### 2.12. Morphological Study of the Micropellets Using Scanning Electron Microscopy (SEM)

Morphological characterization of the micropellets was done by taking scanning electron micrograph in JEOL (Japan), JSM Model 5200 Scanning Electron Microscope. The samples were initially coated to 200 Å thickness with gold-palladium using Pelco Model 3 sputter coater, prior to microscopy.

### 2.13. *In Vitro* Drug Release Studies of Frusemide Loaded Calcium Alginate Micropellets

The USP rotating, paddle dissolution rate apparatus (Veego, Mumbai, India), was used to study drug release from the micropellets. The rotation of the paddles was fixed to 50 rpm, and temperature was kept at 37 ± 2°C throughout the experiment. At these specified time intervals (0.5, 1, 2, 3, 4, 5, 6, 7, 8, and 9 hrs.), a fixed volume of sample (10 mL) was withdrawn from the dissolution medium (phosphate buffer of pH 6.8) and substituted by equal volume of fresh medium. The withdrawn samples were diluted suitably, and the drug contents in the samples were determined by using SHIMADZU UV-VIS 2400 spectrophotometer at 277.5 nm. The tests were performed in triplicate, and mean value was taken for further calculation.

### 2.14. *In Vivo* Performance of the Prepared Sustained Release Calcium Alginate Micropellets

To assess the pharmacological (diuretic) activity through *in vivo* performance of the prepared sustained release calcium alginate micropellets containing frusemide (F9), it was compared with conventional frusemide tablet (LASIX), as standard diuretic agent, after administering it orally to male Wistar rats. The purpose was met by estimating the efficacy of the test formulation (F9) in producing diuresis for more than 8 hours when compared to a standard diuretic agent and a placebo control (normal saline, 0.9% w/v) using urine analysis data of the animals. Modified Lipschitz [15, 16] test model was employed to estimate excretion of water (H_2_O), sodium (Na), and potassium (K) in test animals after administration of the test formulation and compared with the results obtained from the animals treated with high dose of urea. The entire procedure followed noninvasive method, and necessary permission was obtained from the Institutional Animal Ethics Committee (vide sanction of proposal no. HPI/07/60/IAEC/0004). 3 groups consisting of 5 animals, in each, of Wistar albino rats were kept unfed without water for 15 hour. The test group (Group T) received the test formulation (F9) at a dose of 50 mg/kg body weight in 5 mL water orally. Additionally, 5 mL of normal saline solution per 100 g body weight was given by gavages. The standard group (Group S) received the standard tablet (LASIX, 40 mg) admixed, at the same dose of test formulation in normal saline. The control group (Group C) received only 5 mL of normal saline solution per 100 g body weight. Excretion of urine was recorded after 5 and 24 hours (Table 8) to have a clear estimation of the duration of the diuretic effect. Sodium and potassium contents in the collected urine were determined (Table 9, Figure 8) by using Flame Photometer, Model Type 121, Systronics, India.

The Lipschitz quotients for urine volume and sodium ion excretion for both standard and test samples were tabulated in Table 10.

## 3. Result and Discussion

### 3.1. Micro Pellet Morphology and Particle Size Analysis

After drying, the moist micropellets were kept in an oven for 6 hours at 60°C, and the resultant dry micropellets were physically evaluated which were discoid in shape rather than purely spherical. With the increase in polymer load, namely, the formulations F7, F8, and F9, more sphericity were obtained. The micropellets containing only sodium alginate (formulations F1, F4, and F7) were off-white in colour whereas with the addition of Acrycoat E30D light yellowish micropellets resulted. From the particle size analysis of frusemide loaded calcium alginate micropellets as observed form Table 3, it is evident that the size range of all the formulations ranged between 600 *μ*m and 850 *μ*m. According to the results of the size range, it was technically proved that the term micropellets for the formulation had been correctly coined. They could also be classified under microspheres as the size range of microspheres ranges 5 *μ*m–1000 *μ*m. The micropellets of first three formulations having low level (1% w/v) of sodium alginate showed particles of least size range compared to the remaining six, though formulations F7 and F8 showed similar results. Formulations F3, F6, and F9 which contain high level (4% w/w) of Acrycoat E30D showed steady increase in particle size along with different levels of sodium alginate. 

### 3.2. Rheology of Micropellets of Frusemide

All the formulations showed angle of repose in a range 16°–23°. On interpreting the result with the values of Table 3, it could be inferred that all formulations of frusemide micropellets were free flowing and while tableting them or encapsulating in capsule shell, and lubricants need not to be added.

### 3.3. Drug Content and Drug Entrapment Efficiency (DEE)

 From the results presented in Table 3, it was observed that ionotropic gelation technique produced micropellets of high encapsulation efficiency. The DEE value varies within a very short range from 91 to 99% among all the nine formulations indicating very small amount of drug loss during the process. The formulations F1, F2, and F3 with low level of sodium alginate (1% w/v) showed marginally higher entrapment when compared to the other two levels. This may be explained by the fact that at higher concentration sodium alginate forms a rigid matrix with Calcium Chloride preventing incorporation of drug during the curing period in the calcium chloride solution. However the study evidenced that sodium alginate and Acrycoat E30D do not have much significant effect in the drug content of the formulations, since the drug contents were within ±5% of the labelled potency of the drug in all the formulations.

### 3.4. Loose Surface Crystal (LSC) Analysis

The values of LSC were an important parameter giving an indication of the amount of drug available on the surface of the micropellets for immediate absorption and to elicit quick onset of action. The data presented in Table 3 showed that very small amount of drug ranging from 0.604% to 3.549% with respect to total entrapped drug is available loosely on the surface of the micropellets. It was also observed that with the increase in the concentration of Acrycoat E30D, there was a gradual decrease in the value of %LSC. The formulations prepared without secondary polymer (Acrycoat E30D), namely, F1, F4, and F7, reflected much higher amount of drug loosely available on the surface. 

### 3.5. Disintegration Study

The results of disintegration study revealed that the ionic character of the polysaccharides showed pH-dependent disintegration of the micropellets. In calcium alginate pellets, the carboxyl groups in the alginate moiety got ionized in the higher pH, thereby repelling each other. As a result, fluid was drawn inside the pellets producing swelling, and ultimately the micropellets busted out. With the increase in the proportion of polymer and copolymer (F6 to F9) the disintegration time was delayed, as evidenced from Table 3, due to dense network formation which hindered withdrawal of fluid, hence swelling of the pellets. Formulation F9 showed exceptionally high (>2 hr) disintegration time extended beyond the testing time (2 hr) and hence could not be recorded. On keeping this particular formulation for overnight, it was found to be disintegrated and dispersed in the medium. The data obtained also had given an indication of the sustained effect produced by the polymers in releasing the drug in the dissolution medium.

### 3.6. FTIR Analysis of Frusemide Micropellets

**Figure 2:**
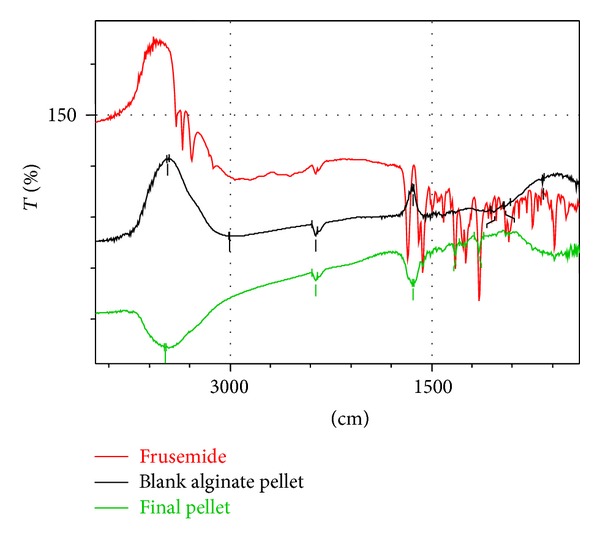
IR spectra of frusemide, blank alginate pellets and final micropellets.

The IR spectra of the materials obtained were presented in Figure 2. From the infrared spectra it was clearly evident that there were no interactions of the drug, frusemide, with the excipients used, namely, sodium alginate and Acrycoat E30D. The main peaks in the spectrum of the drug frusemide like 1143.83 and 1323.21/cm for S=O bond, 1674.27/cm for C=O bond, 3487.42/cm for N–S bond, and 582 for C–Cl bond remained undisturbed in the final formulation. This had proven the fact that there was no potential incompatibility of the drug with all the polymers used in the formulations. Hence, the formula for preparing frusemide loaded calcium alginate microspheres could be reproduced in the industrial scale without any apprehension of possible drug-polymer interactions.

### 3.7. Drug-Polymer Interaction Analysis by HPLC

**Figure 3:**
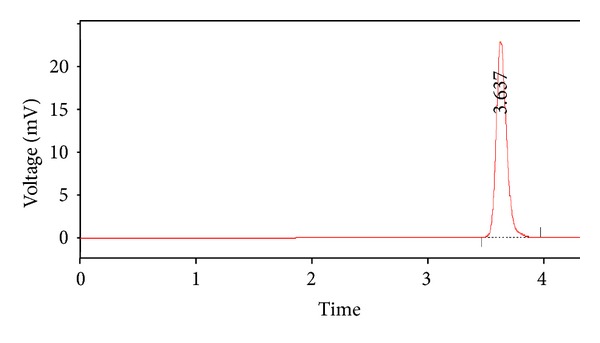
Chromatogram of the standard sample of pure frusemide.

**Figure 4:**
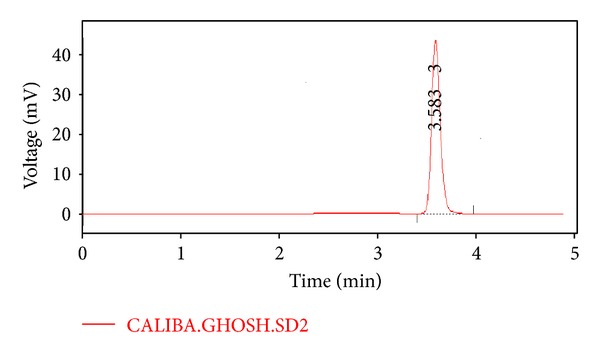
Chromatogram of the Test Sample containing Frusemide and Polymers.

Chromatograms as represented in Figures 3 and 4, the retention time of both standard sample of pure frusemide and test sample containing Frusemide and polymers was found to be very close and the value of *R*_*r*_ ~ 1, signifying no detectable interaction among the drug, frusemide, and the polymers such as calcium alginate and Acrycoat E30D which were present in the formulations. This conclusion were further augments the results obtained from FTIR study.

### 3.8. Scanning Electron Microscopy of Frusemide Micropellets

**Figure 5:**
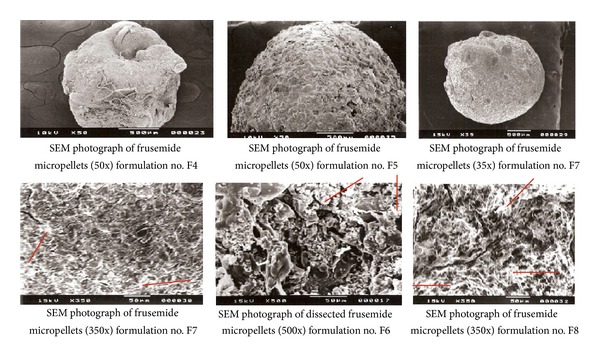
Scanning electron micrographs of Frusemide calcium alginate micro pellets.

 The scanning electron micrograph of the final sets of micropellets presented in Figure 5 showed that all the formulations produced rounded, rough pellets which could be defined to be purely spherical. When studied at higher magnification (350X and 500X) and when the pellets were centrally dissected and the cross-sectional views were captured, the pellets showed dense network of polymers entrapping the drug. The numerous channels and pores visible in the micrograph showed the pathway through which dissolution medium could permeate into the drug-polymer matrix, swell the polymers, and widen the pore diameter which in turns would help the drug molecule to diffuse from the matrix into the dissolution medium. This fact explained the sustained release nature of the formulation. Presence of free drug on the surface of the pellets supported the logic behind the loose surface crystal study reported.

### 3.9. Analysis of *In Vitro* Drug Release Kinetics and Mechanism

**Figure 6:**
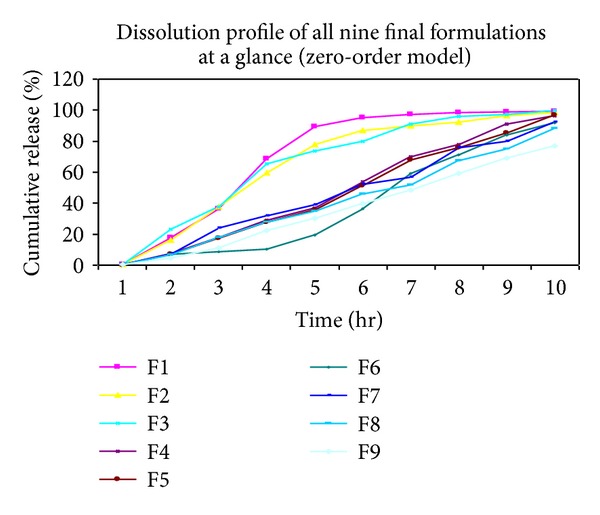
Release profile of frusemide from micropellets at different levels of sodium alginate (−1, 0, +1) following zero-order model.

 The *in vitro* release study unfolded quite an interesting result. Prior to the *in vitro* studies it was expected that at a particular concentration of sodium alginate, with the increase in concentration of Acrycoat E30D, there would be a decrease in the release rate with the extension of the sustaining release of frusemide from micropellets. In order to analyse the drug release mechanism from the micropellets the following mathematical models were studied, namely, zero-order model (Figure 6), first-order model, Higuchi model, and Hixson-Crowell model. The correlation coefficient (*R* ^2^) was used to compare the model equations depicted in Table 5 and to determine the “best fit” model that explains the release kinetics of the drug from the prepared micropellets. The concentrations of the independent variables and the corresponding-dependent factors obtained from experiments of *in vitro* dissolution study were enlisted in Table 6.

Among the three batches (F1, F2, and F3) F2 was found to be best fit with first-order kinetics, and for F1 and F3 the *R* ^2^ values of both Hixson-Crowell and first-order kinetics were in close proximity. Hence it could be inferred that at low concentration of sodium alginate the surface area of micropellets decreased exponentially with the time during the dissolution process. During the agitation of the dissolution process there was no stagnation of dissolved, drug and thus proper sink conditions were maintained. In these three formulations the drug released at any time remained proportional to the residual drug inside the dosage form. Further, when the release profile was divided into two phases to get a more specific release mechanism, it was seen from the *R* ^2^ value (Table 7) that in the phase I the drug release was predominant following the first-order kinetics. Thus, it could be concluded that at low concentration of sodium alginate the micropellets acted as reservoir devices of frusemide which released its content depending on the concentration gradient. In spite of incorporation of Acrycoat E30D as copolymer, they could not form any drug-polymer matrix. 

In the rest six batches (F4–F9), the phase I release followed first-order, except the formulations F3 and F6 which followed the zero-order release with a constant release rate over the time, independent of drug concentration in the system. On the basis of the results obtained, it could be concluded that release of frusemide from the micropellets followed a mixed kinetics, that is, initially first-order kinetics followed by zero-order kinetics. The effect of Acrycoat E30D as release rate controlling polymer was evident from the fact that with increase in the Acrycoat concentration, value of *K*_0_ decreased in every case. At the high level of alginate this fact was more pronounced where the micropellets were found to release the active ingredients at a rate ranging 8–10 mg/hr. 

For all the batches of formulation the release exponent (*n*) in an overall span of 0–9 hr was >1 signifying super-Case II non-Fickian anomalous diffusion transport mechanism. 

The dehydrated hydrogels generally involved the simultaneous absorption of water and desorption of drug via a swelling-controlled diffusion mechanism [17]. Similar to the transport of organic penetrant in glassy polymers, diffusion and swelling in glassy hydrogels generally would not follow a Fickian diffusion mechanism. The slow reorientation of polymer chains in order to accommodate the penetrating solvent molecules leads to a variety of sorption behaviours, particularly when the experimental temperatures were near or below the glass transition temperature of the hydrogel. In cases where sorption process was governed by the rate of polymer relaxation, Case II transport was followed and characterized by linear time dependence in the amount diffused and the penetrating swelling front position resulted. Generally, in most systems, the intermediate situation, termed as non-Fickian or anomalous diffusion, prevailed, whenever the rates of diffusion and polymer relaxation were comparable. On segregating the study in two phases phase I and phase II, (Table 7) this phenomenon of drug release was very much in proximity with the theory as it was seen that in the initial phase *n* ≫ 1 reflecting the non-Fickian release. With time, water penetrated into the hydrogel matrix containing dispersed drug, and the polymer chains had taken up a finite amount of time to rearrange to an equilibrium state in order to accommodate the penetrating solvent. On significant hydration drug release tends to be linear with time giving (*n* ~ 1) signifying zero-order transport mechanism. 

Thus it can be concluded, that by increasing the polymer mass in the micropellets such drug delivery device could be generated which could retain a constant geometry with a constant release rate of drug following zero-order kinetic model. The reproducibility of the polymers to produce micropellets of similar release mechanism was optimized statistically. 

### 3.10. Optimization Data Analysis and Validation of Optimization Model

The values of *K*_0_ obtained under the different experimental conditions for all the nine formulations were summarized in Table 6. 

The application of RSM offers, on the basis of parameter estimate, an empirical relationship between the response variable *K*_0_, *X*_120_, *t*_80_, and *n* individually and the test variables under considerations. Quadratic model (partial sum of squares—Type III) was selected for all the RSM studies. By applying multiple regression analysis on the experimental data, the response variable *K*_0_, *X*_120_, *t*_80_, and *n* and the test variables *A* (concentration of sodium alginate % w/w) and *B* (concentration of Acrycoat E30D % w/w) were related by second-order polynomial equations.

**Figure 7:**
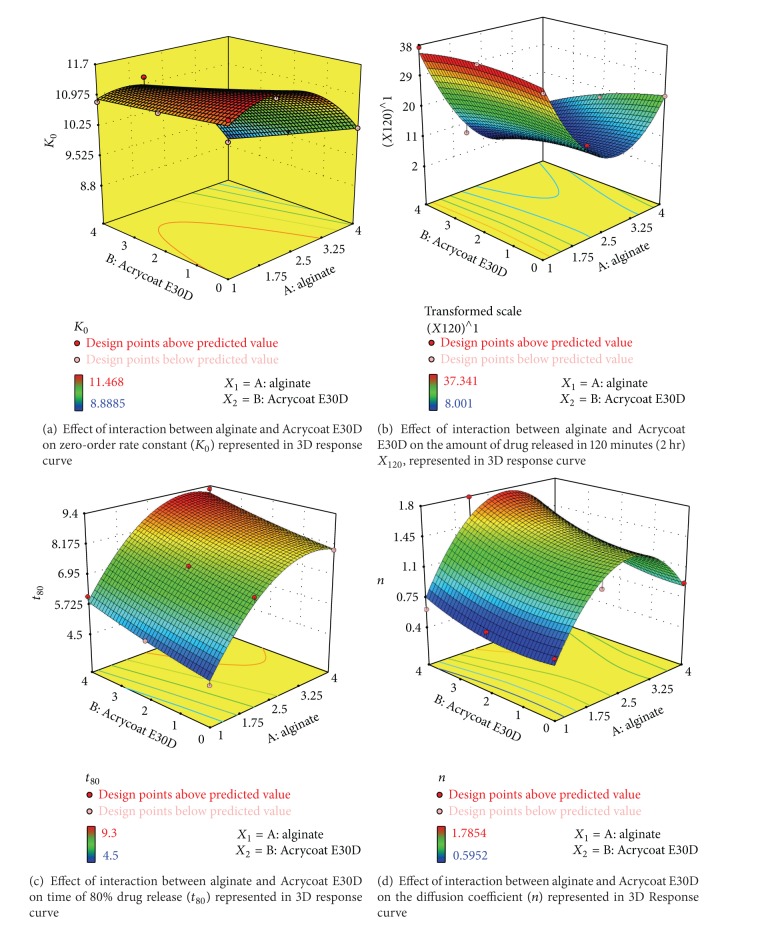
Effect of interaction between alginate and Acrycoat E30D on experimental variables represented in 3D response curves.

**Figure 8:**
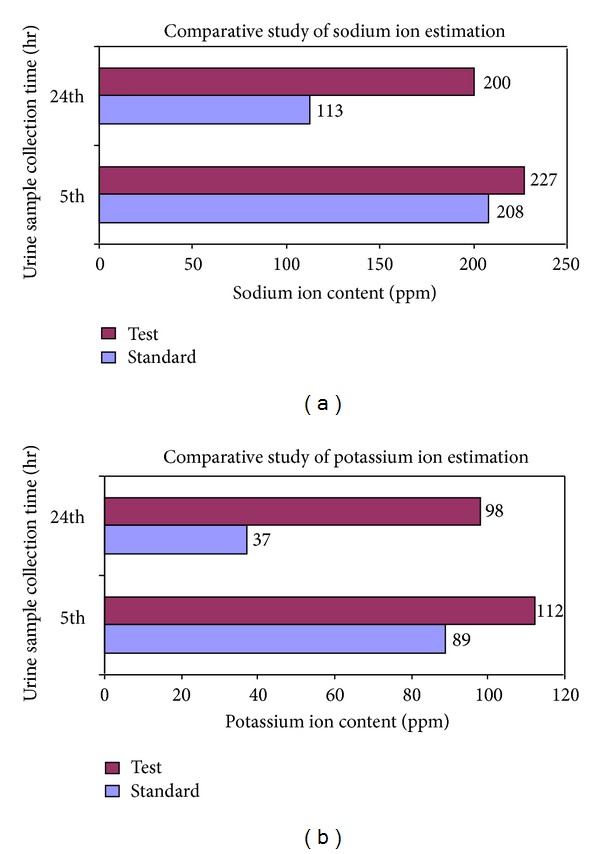
Comparative study of sodium ion and potassium ion estimation among test (micropellets) and standard (Lasix tablet) containing frusemide (40 mg) in Flame Photometer.

The graphical representation of the regression equations called the response surfaces and 3D curves were obtained using the software Design Expert 7.1.2 and were presented in Figure 7.

 The model equation related to zero-order release rate constant (*K*_0_) as response became

(3) Ko=11.11−0.72×A−0.38B−0.23×A×B−0.80×A2+9.683E−003×B2.

A summary of the analysis of variance (ANOVA) for the selected quadratic predictive model is shown in Table 4. Statistical testing of the model was done in the form of analysis of variance (ANOVA) which is required to test the significance and adequacy of the model. Here the ANOVA of regression model demonstrates that the model is highly significant, as evident from the calculated *F* value (40.21) and a very low probability value (*P* < 0.006). The model was found to be adequate for prediction within the range of the variables employed. The *P* values are used as a tool to check the significance of each of the coefficients, which in turn may indicate the pattern of interactions between the variables. The smaller the value of *P*, the more significant the corresponding coefficient. It can be seen from Table 4 that the significant coefficients were *A*  (*P* = 0.0019), *B*  (*P* = 0.0127), and *A* ^2^  (*P* = 0.0105), the *P* values of all of them being small at 5% confidence interval (*P* < 0.0500). The coefficients *A*, *B*, and *A* ^2^ had a negative effect on the zero-order release rates (*K*_0_) of the drug from the prepared micropellets.

The model equation related to amount of drug released in 2 hours (burst effect) *X*_120_ as response became

(4) X120=9.59−9.65×A−3.46×B−3.24×A×B+18.44×A2−1.69×B2.

Here the ANOVA of regression model demonstrates that the model is highly significant, as evident from the calculated *F* value (33.26) and a very low probability value (*P* = 0.0079). The model was found to be adequate for prediction within the range of the variables employed. It can be seen from Table 4 that the significant coefficients were *A*  (*P* = 0.0024), *B*  (*P* = 0.0424), and *A* ^2^  (*P* = 0.0027) the *P* values of all of them being small at 5% confidence interval (*P* < 0.0500). The coefficients *A*, *B*, and *A* ^2^ had a negative effect on the burst release (*X*_120_) of the drug from the prepared micropellets. 

The model equation related to time required for 80% drug release *t*_80_ (hr) as response became

(5) t80=8.17+1.65×A+0.58×B−0.027×A×B−1.31×A2+0.083×B2.

ANOVA of regression model demonstrates that the model is highly significant, as evident from the calculated *F* value (38.12) and a very low probability value (*P* = 0.0065). The model was found to be adequate for prediction within the range of the variables employed. It can be seen from Table 4 that the significant coefficients were *A*  (*P* = 0.0010), *B*  (*P* = 0.0212), and *A* ^2^  (*P* = 0.0145), the *P* values of all of them being small at 5% confidence interval (*P* < 0.0500). The coefficients *A*, *B*, and *A* ^2^ had a negative effect on the time taken to release 80% (*t*_80_) of the drug from the prepared micropellets.

 The model equation related to Peppas diffusion coefficient *n* as response became

(6) n=1.45+0.24×A+0.17×B+0.057×A×B−0.69×A2+0.11×B2.

The ANOVA of regression model demonstrates that the model is insignificant at a confidence interval of 5%, as evident from the calculated *F* value (5.56) and a high probability value (*P* = 0.0943). The model was found to be inadequate for prediction within the range of the variables employed. It can be seen from Table 4 that the significant coefficient is *A* ^2^  (*P* = 0.0232), the *P* value being small at 5% confidence interval (*P* < 0.0500). The coefficients *A*, *B* had no significant effect, and only the term *A* ^2^ had a statistically significant effect on the diffusion coefficient (*n*) of the drug from the prepared micropellets.

In the research work, increased polymer level in the formulations resulted in decreased drug release rates (*K*_0_) and burst effect (*X*_120_) and increased time (*t*_80_) for the release of 80% of the drug from the formulated micropellets. The effect of the primary polymer, sodium alginate, was much more significant statistically (*P* < 0.05) than the copolymer Acrycoat E30D. The combined effect of both the polymer was proportional to the sole effect of sodium alginate. It could also be concluded that the concentration range within 2–4% (w/w) of sodium alginate could give a predictive and reproductive formulation. 

 Though concentrations above 4% were not investigated, it could be assumed from the statistical result that no robust change in the dissolution behaviour would be achieved. On the contrary, higher concentration of polymers would increase the particle size and may also pose mechanical problems during extrusion technique due to high polymer viscosity.

### 3.11. *In Vivo* Evaluation of the Diuresis Followed by Optimized Formulation

The optimized formulation, that is, F9 (4% w/w of both sodium alginate and Acrycoat E30D with 30% drug load), which recorded maximum *in vitro *extended release of the drug over a span of 9 hour, was examined for pharmacological activity through *in vivo* Lipschitz test as the test has been proven to be a standard and a very useful tool for screening of potential diuretics. The Lipschitz value for both standard and test sample was found to be greater than 2.0 at the end of both 5th and 24th hours (Table 10), confirming the positive diuretic effect of both samples. But at the end of the 24th hour, test sample showed significantly (*P* < 0.05) high quotient (7.02) implying high volume of urine being excreted on using the micropellets when compared to the standard tablets (4.63). It also proves the efficacy of the formulation in providing therapeutic effect to the subject. This is further supported by the increase in percentage of volume of urine collected compared to the control group. Though at the end of the 5th hour both standard and test sample showed similar increment (300 and 362.5%) at the end of 24th hour test sample again showed significantly (*P* < 0.05) high increment, 602.1%, whereas the standard showed an increment value of 444.5%. This establishes the fact that the test formulation was successful in sustaining the diuretic effect of the drug till 24 hours. Thus the objective of the research work was partly met from these data.

## 4. Conclusion

One of the primary objectives of the research work was to achieve controlled release micro particulate drug delivery systems in the form of micropellets through the use of a combination of water-soluble polymer such as sodium alginate and a water-insoluble polymer Acrycoat E30D. In the research work, increased polymer level in the formulations resulted in decreased drug release rates (*K*_0_) and burst effect (*X*_120_) and increased time (*t*_80_) for the release of 80% of the drug from the formulated micropellets. The effect of the primary polymer, sodium alginate, was much more significant statistically (*P* < 0.05) than the copolymer Acrycoat E30D. The combined effect was proportional to the sole effect of sodium alginate. All other pharmacokinetic parameters remained fairly unchanged with its incorporation, and by increasing the polymer mass in the micropellets such drug delivery device could be generated which can retain a constant geometry with a constant release rate of drug following zero-order kinetic models. It could also be concluded that the concentration range within 2–4% (w/w) of sodium alginate could give a predictive and reproductive formulation. On the contrary, higher concentration of polymers would increase the particle size and may also pose mechanical problems during extrusion technique due to high polymer viscosity. The *in vivo* study acclaimed that calcium alginate micropellets of frusemide can produce a much greater diuretic effect over an extended period of 24 hours. Hence a single dose of the formulation is sufficient in the management of peripheral edema and ascites in congestive heart failure and as well in the treatment of chronic hypertension, leading to better patient compliance.

## Figures and Tables

**Table:** (a)

Study type	Initial design	Center points	Design model	Runs	Blocks
Factorial	Full Factorial	0	2FI	9	No Blocks

**Table:** (b)

Factor	Name	Units	Type	Low Actual	High Actual	Levels
*A*	Alginate	%w/w	Categoric	1%	4%	3
*B*	Acrycoat E30D	%w/w	Categoric	0%	4%	3

**Table:** (c)

Response	Name	Units	Observn.	Analysis	Min.	Max.	Mean	SD	Ratio
Y1	*K*_0_	mg/hr	9	Factorial	8.89	11.47	10.64	0.82	1.29
Y2	*X*_120_	mg	9	Factorial	8.00	37.34	22.51	10.7	4.67
Y3	*t*_80_	hr	9	Factorial	4.50	9.30	7.12	1.47	2.07
Y4	*n*		9	Factorial	0.60	1.79	1.01	0.37	3.00

The design matrix Evaluation for factorial 2FI model.

Degrees of freedom (dF) for evaluation, model: 8, residuals: 0, lack of fit: 0, pure error: 0, and total: 9.

**Table 2:** Full 3^2 ^factorial design for the formulation of frusemide loaded calcium alginate micropellets.

Batch no./formulation code	Sodium alginate	Acrycoat E30D
F1	−	−
F2	−	0
F3	−	+
F4	0	−
F5	0	0
F6	0	+
F7	+	−
F8	+	0
F9	+	+

Level	Sodium alginate (%w/w)	Acrycoat E30D (%w/w)

+	4.0	4.0
0	2.0	2.0
−	1.0	0.0

Constants: calcium chloride concentration—5%w/v, drug loading concentrations—30%w/w.

**Table 3:** Evaluation result of physicochemical parameter of frusemide calcium alginate micropellets.

Formulation code	Mean diameter*	Angle of repose (*θ*)**	% Moisture content**	Drug entrapment efficiency (%)**	LSC (%) with respect to gross wt. of the micropellets	LSC (%) with respect to entrapped drug	Disintegration time (min)**
	(*µ*m ± SD)						
F1	608.14 ± 0.39	16.76 ± 0.54	1.71 ± 0.81	95.94 ± 0.63	2.271	2.367	24 ± 4.53
F2	655.90 ± 1.03	18.06 ± 0.96	1.59 ± 0.76	99.02 ± 0.82	1.943	1.962	36 ± 5.21
F3	694.85 ± 0.72	20.11 ± 0.88	1.97 ± 0.32	99.47 ± 0.91	1.677	1.686	52 ± 4.68
F4	608.16 ± 0.59	18.32 ± 0.79	1.48 ± 0.48	94.48 ± 0.48	3.353	3.549	42 ± 3.85
F5	760.89 ± 0.51	20.56 ± 1.03	1.44 ± 0.56	93.44 ± 0.56	2.214	2.369	64 ± 6.11
F6	782.78 ± 0.36	21.24 ± 1.97	2.23 ± 0.68	91.23 ± 0.68	1.429	1.567	97 ± 4.37
F7	632.1 ± 0.73	19.52 ± 1.21	1.67 ± 0.33	96.21 ± 0.37	1.882	1.956	73 ± 5.24
F8	693.36 ± 0.56	21.39 ± 1.07	1.42 ± 0.54	97.76 ± 0.56	0.913	0.729	115 ± 5.98
F9	841.65 ± 0.48	22.56 ± 0.77	2.32 ± 0.64	98.37 ± 0.77	0.594	0.604	>120

*Results shown are “mean ± SD,” *n* = 6 for mean diameter; signifies value < 0.01%. **Results shown are “mean ± SD,” *n* = 3.

**Table 4:** Summary of ANOVA for the response parameters.

Source	Sum of squares	Degree of freedom dF	Mean square	*F* value	*P* value prob. > *F*	Effect
For “*K*_0_”						
Model	5.91	5	1.18	40.21	0.0060	significant
*A*: alginate %w/w	3.13	1	3.13	106.59	0.0019	
*B*: acrycoat E30D %w/w	0.83	1	0.84	28.70	0.0127	
*AB*	0.23	1	0.23	7.77	0.0685	
*A* ^2^	0.97	1	0.97	32.96	0.0105	
*B* ^2^	1.875*E* − 004	1	1.875*E* − 004	6.379*E* − 003	0.9414	
Residual	0.088	3	0.029			
Correction total	6	8				

For “*X*_120_”						
Model	1012.2	5	202.44	33.26	0.0079	significant
*A*: alginate %w/w	558.39	1	558.39	91.74	0.0024	
*B*: acrycoat E30D %w/w	70.4	1	70.4	11.57	0.0424	
*AB*	43.53	1	43.53	7.15	0.0754	
*A* ^2^	517.97	1	517.97	85.10	0.0027	
*B* ^2^	5.74	1	5.74	0.94	0.4032	
Residual	18.26	3	6.09			
Correction total	1030.46	8				

For “*t*_80_”						
Model	19.15	5	3.83	38.12	0.0065	significant
*A*: alginate %w/w	16.34	1	16.34	162.55	0.0010	
*B*: acrycoat E30D %w/w	1.99	1	1.99	19.76	0.0212	
*AB*	2.176*E* − 003	1	2.176*E* − 003	0.03	0.8743	
*A* ^2^	2.63	1	2.63	26.12	0.0145	
*B* ^2^	0.014	1	0.014	0.14	0.7348	
Residual	0.30	3	0.10			
Correction total	19.46	8				

For “*n*”						
Model	1.11	5	0.22	5.56	0.0943	Not significant
*A*: alginate %w/w	0.33	1	0.33	8.38	0.0627	
*B*: acrycoat E30D %w/w	0.17	1	0.17	4.15	0.1345	
*AB*	0.013	1	0.013	0.33	0.6043	
*A* ^2^	0.73	1	0.73	18.43	0.0232	
*B* ^2^	0.025	1	0.025	0.63	0.4864	
Residual	0.12	3	0.04			
Correction total	1.23	8				

*A* and *B* represent the main effects (factors); *A* ^2^ and *B* ^2^ are the quadratic effect; *AB* is the interaction effect.

**Table 5:** Correlation coefficients (*R* ^2^) of different plots for overall (0–9 hr) release kinetics of frusemide from prepared micropellets.

Kinetic models	Correlation coefficients (*R* ^2^ values)								
	Level (−1)			Level (0)			Level (+1)		
	F1	F2	F3	F4	F5	F6	F7	F8	F9
Zero order	0.8091	0.8749	0.8949	0.9918	0.9931	0.9397	0.9918	0.9945	0.9945
First order	0.9240	0.9824	0.8916	0.8678	0.8265	0.8531	0.8328	0.8933	0.9498
Higuchi	0.9003	0.9495	0.9599	0.9653	0.9638	0.8666	0.9750	0.9644	0.9606
Hixson-Crowell	0.9400	0.9819	0.9911	0.9473	0.9314	0.9025	0.9303	0.9485	0.9756

**Table 6:** Independent formulation variables and their responses.

Formulation code	Sodium alginate conc. %(w/w)	Acrycoat E30D conc. %(w/w)	*K*_0_ (mg/hr)	*X*_120_ (mg)	*t*_80_ (hr)	*n*
F1	1	0	10.865	34.95	4.5	1.7429
F2	1	2	10.209	37.34	5.2	1.7309
F3	1	4	10.299	37.341	6.1	1.4378
F4	2	0	9.3247	16.988	7.2	1.8142
F5	2	2	9.0388	16.183	7.6	2.3807
F6	2	4	7.9428	8.001	7.8	1.8064
F7	4	0	7.5642	23.173	8	1.6173
F8	4	2	6.9212	17.382	8.4	1.6074
F9	4	4	6.1069	11.194	9.3	1.5608

**Table 7:** Korsmeyer-Peppas model fitting release rate constants (*K*_KP_), correlation coefficient (*R* ^2^), and release exponent (*n*) of frusemide from micropellets with different levels of alginate level (−1, 0, and +1).

Korsmeyer-Peppas model	Overall release (0–9 hr)			PHASE I (0–2 hr)			PHASE II (2–9 hr)		
Formulation code	*K*_KP_	*R* ^2^	*n*	*K*_KP_ I	*R* ^2^ I	*n* I	*K*_KP_ II	*R* ^2^ II	*n* II
F1	0.6731	0.7589	1.7429	0.7173	0.8535	3.6857	1.4996	0.7718	0.5952
F2	0.6529	0.7663	1.7309	0.7107	0.8682	3.7106	1.4664	0.8925	0.6015
F3	0.8685	0.7749	1.4378	0.9114	0.8359	2.96	1.4686	0.9026	0.6005
F4	0.4384	0.8909	1.8142	0.4954	0.895	3.1443	0.9029	0.9924	1.1637
F5	0.0197	0.7984	2.3807	0.1278	0.8487	4.8791	0.8831	0.9935	1.1705
F6	0.3049	0.9127	1.8064	0.3878	0.7992	2.5957	0.3002	0.9606	1.7854
F7	0.567	0.9004	1.6173	0.6205	0.9558	2.8874	1.0778	0.9844	0.9125
F8	0.5268	0.9177	1.6074	0.5699	0.9341	2.6683	0.9295	0.995	1.0477
F9	0.4982	0.969	1.5608	0.5086	0.9527	2.0875	0.719	0.9928	1.2522

**Table 8:** Lipschitz test values of the collected urine sample.

Sl. no.	Group	Treatment	Mean volume of urine collected (mL)		Lipschitz value (T or S/C)		% Increase in Volume of Urine ((T or S − C)/C) × 100	
			5th hour (mL)	24th hour (mL)	5th hour	24th hour	5th hour (%)	24th hour (%)
1 2 3 4 5	Control (C)	Normal saline 5 mL/100 g body wt.	1.8 ± 0.92	4.8 ± 1.29	1.00	1.00	—	—

6 7 8 9 10	Standard (S)	Pure frusemide 50 mg/kg p.o.	7.2 ± 1.37	22.2 ± 3.61	4.00	4.63	300.0	444.5

11 12 13 14 15	Test (T)	Frusemide micropellets 50 mg/kg p.o.	9.8 ± 2.06	33.7 ± 4.11	5.45	7.02	362.5	602.1*

Values are expressed ± SEM

^“∗”^Indicates (*P* < 0.05).

**Table 9:** Flame photometry mean readings of sodium and potassium ions in urine sample from Groups C, S, and T after 5 and 24 hours.

Sl. no.	Group	Treatment	Na+ ion		K+ ion	
			5th hour	24th hour	5th hour	24th hour
1 2 3 4 5	Control (C)	Normal saline 5 mL/100 g body wt.	24	28	21	11

6 7 8 9 10	Standard (S)	Pure frusemide 50 mg/kg p.o.	69	44	25	14

11 12 13 14 15	Test (T)	Frusemide micropellets 50 mg/kg p.o.	74	67*	30	27*

Values are expressed ± SEM

^“∗”^Indicates (*P* < 0.05), *n*=5.

**Table 10:** Results of the *in vivo* parameters studied for both standard (S) and test (T) samples after 5 and 24 hours.

Sl. no.	Parameter	Standard sample (Lasix tablet)		Test sample micropellets (F9)	
		5th hour	24th hour	5th hour	24th hour
1	Lipschitz quotient for urine volume	4.00	4.63	5.45	7.02*
2	% Increase in volume of collected urine	300.0	444.5	362.5	602.1*
3	Lipschitz quotient for Na+ excretion	5.78	2.17	6.31	4.00*
4	Lipschitz quotient for K+ excretion	1.27	1.61	1.60	4.26

*Indicates (*P* < 0.05) *n* = 5.
